# Evaluation of early coronary graft patency after coronary artery bypass graft surgery using multislice computed tomography angiography

**DOI:** 10.1186/1471-2261-9-53

**Published:** 2009-12-08

**Authors:** Hosseinali Bassri, Farzad Salari, Fereidoun Noohi, Marzieh Motevali, Seifollah Abdi, Nader Givtaj, Kamal Raissi, Majid Haghjoo

**Affiliations:** 1 Department of Interventional Cardiology, Rajaie Cardiovascular Medical and Research Center, Iran University of Medical Sciences, Tehran, Iran; 2 Department of Radiology, Rajaie Cardiovascular Medical and Research Center, Iran University of Medical Sciences, Tehran, Iran; 3 Department of Cardiovascular Surgery, Rajaie Cardiovascular Medical and Research Center, Iran University of Medical Sciences, Tehran, Iran; 4 Department of Pacemaker and Electrophysiology, Rajaie Cardiovascular Medical and Research Center, Iran University of Medical Sciences, Tehran, Iran

## Abstract

**Background:**

Coronary artery bypass graft (CABG) surgery is the standard of care in the treatment of advanced coronary artery disease, and its long-term results are affected by the failure of bypass grafts. The aim of the present study was to evaluate the early patency rate in coronary bypass grafts.

**Methods:**

A total of 107 consecutive patients who underwent CABG were included in this study. Early graft patency was evaluated via computed tomography (CT) angiography in the first week after surgery.

**Results:**

There were a total of 366 grafts, comprised of 250 venous grafts and 116 arterial grafts. Multi-slice CT detected acute graft occlusions in 32 (8.7%) of all the grafts, including 26 (10%) of the 250 venous grafts and 6 (5%) of the 116 arterial grafts. The patency rates obtained were 97.3% for the left internal mammary (IMA) grafts, 50% for the radial artery grafts, and 50% for the right IMA grafts. Additionally, 107 (96.4%) grafts to the left anterior descending artery (LAD) were classified as patent, whereas 1 (30%) of the 3 grafts in the left circumflex (LCX) region and 1 (50%) of the 2 grafts in the right coronary artery (RCA) territory were found to be occluded. In the venous category, 8 (13.7%) of the 58 grafts to LAD were found to be occluded. In the LCX region, 9 (8.5%) of the 106 grafts were classified as occluded, while the remaining 97 (91.5%) grafts were patent. The venous grafts to RCA were occluded in 9 (10.4%) of the 86 grafts. Amongst the multiple preoperative, intraoperative, and postoperative factors, pump time was significantly longer in the patients with occluded grafts than in those with patent grafts (P = 0.04).

**Conclusion:**

The IMA grafts had the highest early patency rate amongst the coronary bypass grafts. However, the other arterial grafts were associated with a high rate of acute occlusions.

## Background

Coronary artery bypass graft (CABG) surgery is the standard of care in the treatment of advanced coronary artery disease. CABG provides excellent short- and intermediate-term results in the management of stable coronary artery disease; its long-term results are, however, affected by the failure of venous grafts [1]. Early occlusions (before hospital discharge) affect the short-term results, and they tend to occur in 8 to 12 percent of venous grafts. The recognition of the prevalence of acute graft occlusions and reconstruction of these factors are effective in CABG results [1].

Multislice CT (MSCT) scanners combine a high spatial resolution with the ability to demonstrate the anatomy through volume-rendered images, thus producing a more sensitive evaluation than does conventional or spiral CT. The efficacy of 16-slice CT for the detection of bypass graft occlusions has been demonstrated in several studies [2-6]. Therefore, the aim of this study was to evaluate the early patency rate of coronary grafts following CABG using MSCT.

## Methods

Between March 2007 and March 2008, 107 consecutive patients (81 men, 26 women; mean age, 60 ± 9 years) who underwent CABG were included in this study. The patients underwent 16-slice CT angiography one week after CABG. The study protocol was approved by the institution's ethics committee, and written informed consent was obtained from all the patients. The exclusion criteria were serum creatinine > 1.5 mg/dL, allergy to contrast material, hyperthyroidism, and inability to give informed consent

### Multidetector CT angiography protocol

The patients were scanned using a 16-section multidetector CT scanner (SOMATOM Sensation 16, Siemens, Forchheim, Germany). The patients were positioned in the gantry supine and feet first, with the electrocardiographic leads placed on the anterior thorax to enable a retrospectively gated scan. The scan parameters were 140 kV, 0.4-second rotation speed, 400 mA, and 10 × 0.75 detectors. The pitch, which was dependent on the heart rate, averaged 0.3. The CT system automatically recommended a pitch value to optimize the temporal resolution by the number of sectors reconstructed from each scan. The scans were performed in the caudal to cephalic direction, with a scan range from the thoracic inlet through the lung bases. The proximal subclavian arteries were also included. The patients were familiarized with the protocol by practicing the examination, including breath-holding, in advance. Beta-blockers (propranolol or esmolol) were injected intravenously for heart rates exceeding 70 beats per minute, unless underlying contraindications such as asthma were present. A nonionic, iodinated, low-osmolar contrast medium was injected intravenously in doses ranging from 120 to 150 mL, without direct variations with respect to the patients' weight. In addition, 20 ml saline flush (saline chaser) was used to optimize the graft visualization.

### Data analysis

The variables are expressed as mean ± SD for the continuous variables and as absolute or relative frequencies for the categorical variables. The chi-square test was used for the categorical data and the Fisher exact test for cell counts less than five. The patients' characteristics were compared by means of Student's *t*-test in case of the continuous variables. Otherwise, a non-parametric test of Mann-Whitney's U-test was employed. A two-tailed P < 0.05 was considered statistically significant. The software SPSS version 15.0 (SPSS Inc., Chicago, IL, USA) was utilized for the statistical analysis.

## Results

The study population consisted of 107 consecutive patients with CABG who completed the MSCT protocol. A total of the 366 grafts, consisting of 250 venous grafts and 116 arterial grafts were evaluated, all of which could be assessed for patency and occlusion using 16-slice CT. Six (5.6%) patients had 2 grafts, 54 (50.5%) had 3 grafts, 43 (40.2%) had 4 grafts, and 4 (3.7%) had 5 grafts. The multi-slice CT detected acute graft occlusions in 32 (8.7%) of all the grafts. The mean heart rate of the patients was 63 ± 4 beats/min. Of the 107 patients, 10 had atrial fibrillation and 12 experienced frequent atrial extrasystoles. Beta-blocker administration was required in 95% of the patients to decrease the heart rate to the desired level.

### Patency rate of arterial grafts

Of the 116 arterial grafts, 111 (96%) grafts were anastomosed to the left anterior descending artery (LAD). The arterial grafts to LAD consisted of 110 left internal mammary artery (LIMA) grafts and 1 radial artery (RA) graft. The remaining arterial grafts included 2 right internal mammary arteries (RIMA) to the right coronary artery (RCA) and 3 RAs to the left circumflex artery (LCX) grafts. According to MSCT, 6 (5%) of the 116 arterial grafts were classified as occluded and 110 (95%) were classified as patent; moreover, 3 (2.7%) of the 110 LIMA grafts, 2 (50%) of the 4 RA grafts, and 1 (50%) of the 2 RIMA grafts were occluded. One-hundred and seven (96.4%) grafts to the LAD were classified as patent without stenosis and 4 (3.6%) grafts were classified as occluded. One (33%) of the 3 arterial grafts in the LCX region and one (50%) of the 2 bypass grafts to RCA were found to be occluded (Table 1).

**Table 1:** Patency rate in coronary grafts

Graft	Patency rate, n (%)
LIMA-LAD	3/110 (97.3%)
RA-LAD	0/1 (0%)
RA-LCX	2/3 (67%)
RIMA-RCA	1/2 (50%)
SVG-LAD	50/58 (86.3%)
SVG-LCX	97/106 (91.5%)
SVG-RCA	77/86 (89.6%)

LIMA = left internal mammary artery; RA = radial artery; LCX = left circumflex; RIMA = right internal mammary artery; RCA = right coronary artery; SVG = saphenous vein graft

### Patency rate of venous grafts

Of the 250 venous grafts, 58 were anastomosed to LAD, 106 to LCX, and 86 to RCA. The acute occlusions were detected in 26 (10.4%) venous grafts. According to MSCT, 8 (13.7%) of the venous grafts to LAD were found to be occluded. In the LCX region, 9 (8.5%) of the 106 grafts were classified as occluded, whereas the remaining 97 (91.5%) grafts were patent. The venous grafts to RCA were occluded in 9 (10.4%) of the 86 grafts (Table 1).

### Effect of preoperative, intraoperative, and postoperative factors on graft patency

Mean age, sex ratio, presenting symptom (unstable vs. stable angina), preoperative left ventricular ejection fraction, major coronary risk factors, and coronary artery anatomy were similar between the patients with patent and occluded coronary grafts. In addition, the patency rate of the coronary grafts was comparable irrespective of the surgical technique (on-pump vs. off-pump), need for packed cell transfusion, and postoperative cardiac arrest. However, pump time was significantly longer in the patients with occluded grafts than in those with patent grafts (Table 2).

**Table 2:** Comparison of baseline variables in patients with patent and occluded coronary grafts.

Variable	Graft patent (n = 80)	Graft occluded (n = 27)	P-value
Age (years)	59 ± 9.6	60 ± 8.3	0.63
Male	61 (76)	20 (74)	0.80
Number of native vessels involved	2.8 ± 0.37	2.9 ± 0.19	0.20
Total graft number	3.4 ± 0.65	3.4 ± 0.69	0.83
Diabetes	25 (31)	11 (41)	0.37
Dyslipidemia	30 (37.5)	12 (44.4)	0.52
Smoking	25 (31)	5 (18.5)	0.20
Hypertension	33 (41)	10 (37)	0.70
Unstable angina before surgery	41 (51)	14 (52)	0.71
Preoperative LVEF	44 ± 8.5	46 ± 6.3	0.17
On-pump vs. off-pump CABG	76 (95)/4 (5)	24 (89)/3 (11)	0.27
Pump time (minutes)	102 ± 32.5	120 ± 43	0.046
Postoperative LVEF	43 ± 8	41 ± 9	0.27
Packed cell transfusion	56 (70)	19 (69.4)	0.98
Postoperative cardiac arrest	2 (2.5)	1 (3.7)	0.74

Values are n (%) or mean ± SD.

LVEF = left ventricular ejection fraction; CABG = coronary artery bypass graft

## Discussion

This prospective evaluation was conducted to determine the acute coronary graft patency rate in CABG patients using 16-slice CT angiography. The major findings of this study are as follows:

(1) the saphenous vein grafts had an overall patency rate of 90% (86.3%-91.5% depending on the grafted vessel); (2) the early patency rate of the arterial grafts was 95%, including 97.3% for the LIMA grafts, 50% for the RA grafts, and 50% for the RIMA grafts.

The present results confirm an early patency rate of > 90% in coronary bypass grafts [7]. This study also demonstrates that 16-slice CT consistently provides high-quality angiograms of bypass grafts that accurately delineate the presence of graft occlusions or stenoses irrespective of the presence of sinus rhythm or arrhythmias in these patients. Five studies have thus far reported on the accuracy of MSCT to detect graft occlusions/stenoses. The foregoing studies, having evaluated 225 patients via MSCT, compared the results with those of conventional angiography and reported a sensitivity of 85.7-100% and specificity of 94-100% for MSCT [4-9]. A higher sensitivity (100%) and specificity (98-100%) was reported for bypass graft occlusion [3-5,9]. These discrepancies in the sensitivity and specificity of 16-slice CT may be explained by the differences in the timing of MSCT after CABG with consequent discrepancies in the patency rate.

A further elucidation of the efficacy and safety of MSCT requires the results of early coronary graft patency using conventional angiography. Goldman et al. reported an early patency rate (1 week after surgery) of 95% for saphenous vein grafts (SVGs) and 99% for IMA grafts [10]. If an SVG or IMA graft was patent at 1 week, that graft had a 68% and 88% chance, respectively, of being patent at 10 years. Shimokawa and colleagues evaluated the SVG graft patency 11.8 ± 10.4 days after CABG using conventional coronary arteriography and similarly reported an early patency rate of 95.7% [11]. In another study, Puskas et al. reported a patency rate of 100% for IMA grafts [12]. An angiographic follow-up of 5, 065 grafts showed an early patency rate of 88% for SVGs and 95% for LIMA grafts. Taken together, the invasive angiography of coronary grafts is a technically demanding and time consuming inpatient procedure with a small but significant risk of major complications. MSCT provides a highly specific means of detecting CABG patency with similar sensitivity and specificity in an outpatient setting.

The ideal of total arterial revascularization has been based on the assumption that arterial grafts have a superior long-term patency compared with venous bypass grafts. Although there were a limited number of non-LIMA arterial grafts, the present data indicate that the RA grafts were associated with a high rate (50%) of acute occlusions. In a coronary angiographic study, Khot et al. reported a similarly high rate of severe stenosis and occlusion in RA grafts (48.7%) compared with LIMA (9.7%) and SVG (36%) grafts [13]. A selective use of RA is, therefore, warranted.

### Limitations of multi-slice CT angiography

The effect of cardiac motion on the visualization of bypass grafts is exclusively related to the bypass grafts to RCA. All interpretations regarding the grafts in the LAD and LCX territories are correct. As a result, heart rate correction is mainly an issue for the artifact-free visualization of the bypass grafts to RCA and native vessels. In our cohort, mean heart rate was 63 ± 4 beats/min. Therefore, we had artifact free visualization of bypass grafts to RCA in all cases. Another important issue is the presence of metal clips, especially in the IMA grafts. However, a major drawback of MSCT is related to the radiation exposure. A lack of major complications, noninvasive nature, high-quality images, and no need for hospitalization may compensate for the higher radiation dose.

## Conclusion

Our data showed that the SVGs had an overall patency rate of 90% and the arterial grafts were patent in 95% of the grafts, including 97.3% for the LIMA grafts, 50% for the RA grafts, and 50% for the RIMA grafts.

## Competing interests

The authors declare that they have no competing interests.

## Authors' contributions

HB, SA, and FN participated in the interpretation of the multislice CT angiograms; HB also participated in the design of the study. FS collected all the clinical, radiographic, and surgical data. MM carried out the multislice CT angiograms and participated in the interpretation of the CT images. NG and KR performed the CABGs and collected the surgical data. MH drafted the manuscript and performed the statistical analyses. All the authors read and approved the final manuscript.

## Pre-publication history

The pre-publication history for this paper can be accessed here:

http://www.biomedcentral.com/1471-2261/9/53/prepub
